# The role of animacy in language production: evidence from bare noun naming

**DOI:** 10.1177/17470218241281868

**Published:** 2024-09-28

**Authors:** Yufang Wang, Jurriaan Witteman, Niels O Schiller

**Affiliations:** 1 Leiden University Centre for Linguistics, Leiden University, Leiden, The Netherlands; 2 Leiden Institute for Brain and Cognition, Leiden University, Leiden, The Netherlands; 3 Department of Linguistics and Translation, City University of Hong Kong, Kowloon, Hong Kong SAR

**Keywords:** Language production, animacy, Mandarin Chinese classifiers, picture-word interference, bare noun naming, N400, P600

## Abstract

According to Levelt’s language production model, to name an object, speakers must first conceptualise and lexicalise the object before its name can be articulated. Conceptualisation is conducted through the semantic network that exists at the conceptual level, with the highly activated concept(s) activating lexical items at the lemma level, that is, lexicalisation. So far, research focused mostly on semantic categories (i.e., semantic interference) but less so on animacy—a concept that is correlated with semantic categories. To investigate the role of this semantic feature in language production, we conducted a picture-word interference study in Mandarin Chinese, varying animacy congruency and controlling for classifier congruency while recording behavioural and electrophysiological responses. We observed an animacy interference effect together with a larger N400 component for animacy-incongruent versus congruent picture-word pairs, suggesting animacy-congruent concepts may be in closer proximity and hence lead to a stronger spreading of activation relative to animacy-incongruent concepts. Furthermore, a larger P600 component was observed for classifier-incongruent versus classifier-congruent picture-word pairs, suggesting syntactically driven processing of classifiers at the lemma level.

## Introduction

To produce a message, speakers must select appropriate words. Among these words are nouns, which have a semantic category associated with them. Semantic categories (e.g., animals, body parts, furniture, etc.) are collections of concepts that share more elementary semantic features such as shape or animacy. Animacy is the “aliveness” of the referent denoted by the noun, and it is considered to be hierarchical, spanning from “human” (animate) to, e.g., “table” (inanimate) (McRae et al., 2005; Stefanovic, 2000). However, for practical reasons, it is commonplace to regard animacy in a binary form, with nouns being either animate (referring to living entities) or inanimate (non-living entities) (Sá-Leite et al., 2021).

A possible way to experimentally study speech production is to employ the picture–word interference (PWI) paradigm (Pellegrino et al., 1977). In PWI, participants are presented with a picture of a target word superimposed with a written distractor word and asked to overtly name the target noun while ignoring the superimposed written distractor word. A critical finding from previous studies is that naming a target word and being presented with a categorically related distractor word (e.g., naming the picture of a “DOG” with “cat” being the distractor) takes longer than naming the same target but being presented with a categorically unrelated distractor word (e.g., “chair”), that is, the semantic interference effect (Bürki et al., 2020; Collins & Loftus, 1975; Greenham et al., 2000; Levelt, 1999).

This finding of semantic interference effects has been accounted for by a prominent theoretical framework of language production developed by Levelt et al. (1999). Levelt’s model assumes three sequential strata of language production: the conceptual/semantic stratum, the lemma stratum, and the phonological word-form stratum. At the conceptual level, the model proposes that holistic nodes (i.e., semantic representations of words) are connected by a semantic network. The links among the nodes in the semantic network result in the spreading of activation from the intended word to other (unintended) words (Bürki et al., 2020; Levelt, 1999; Levelt et al., 1999; Roelofs, 1992, 1993, 1996). As categorically related words are connected with the same superordinate/hyperonym and are therefore located relatively closer to one another in the semantic network compared with categorically unrelated words, a stronger spreading of activation will occur for categorically related words compared with unrelated words (Levelt, 1999; Levelt et al., 1999; Roelofs, 1992, 1993, 1996). Such proximity-driven spreading of activation allows categorically related words but not categorical unrelated words to be passed down (from the conceptual level to the lemma level) (Collins & Loftus, 1975) and then activate their corresponding lemmas. Here, a lemma denotes a modality-free lexical representation intermediating between the conceptual/semantic and phonological strata, including the syntactic information of a lexical item, for example, its syntactic word class (noun, verb, adjective, etc.) or its grammatical gender (masculine, feminine, neuter, etc.). All activated lemmas then compete for the final lexical selection, denoted as lemma-level competition (originating from the conceptual level). The lemma-level competition reflects a semantically driven interference effect, that is, lower naming accuracies and/or longer naming latencies combined with a larger N400 effect (see the “Study of language production: electrophysiology” section) when naming a target picture under categorically related versus unrelated contexts.

However, the construct of a semantic network itself is currently underspecified (Caramazza, 1997; Levelt et al., 1999; Roelofs, 1992, 1993, 1996)—so far, in Levelt’s model, the only assumption about the network is that words of the same semantic category are in closer proximity to each other than words of different semantic categories (Bürki et al., 2020; Levelt, 1999; Levelt et al., 1999; Roelofs, 1992, 1993, 1996). These semantic categories are typically correlated with some semantic features, such as shape and animacy (Hutchison, 2003; McRae et al., 1997). Thus, the observed effects of semantic categories might to some extent reflect the role of these semantic features. That is, words sharing some semantic features might also have their nodes located in relatively closer proximity in the semantic network, similar to words of the same semantic categories.

In previously reported work, longer naming latencies in object naming tasks have been found under shape-congruent relative to incongruent contexts (De Zubicaray et al., 2018; Gauvin et al., 2019), meaning that semantic features indeed play a role in speech production. These results were interpreted as shape-congruent distractor words spreading activation to the lemma level, leading to lemma-level competition, similar to semantic category interference (De Zubicaray et al., 2018; Gauvin et al., 2019). However, these studies did not specify how overlap in shape results in the interference effect from a semantic network perspective. We posit that the shape congruency effect occurs because shape-congruent words have their nodes in closer proximity than shape-incongruent words in the semantic network. However, it remains unclear whether the results of the semantic feature “shape” can be generalised to other semantic features considering that animacy is a more intricate feature than shape.

In this study, we investigated the role of “animacy” in language production in Mandarin Chinese. The semantic features (e.g., shape and animacy) of a noun in Mandarin Chinese, to some extent, determine the choice of its compatible classifiers (11th edition, Linguistics Institute of the Chinese Academy of Social Sciences, 2011). For instance, an entity being animate, such as 苍蝇 (cang1ying2, “fly”) or 大象 (da4xiang4, “elephant”), results in a large chance of being compatible with classifiers such as 只 (zhi1) and/or 头 (tou2) (Liu et al., 2019). The classifier-incongruent conditions in PWI have been reported to have a significantly more negative N400 effect (see the “Study of language production: electrophysiology” section for detailed information on electrophysiological components) but no significant behavioural difference from classifier congruent conditions (Wang et al., 2019). This reported role of classifiers in electrophysiological responses makes studying the animacy effect in Mandarin Chinese slightly more complex than in other languages (Liu et al., 2019; Wu & Bodomo, 2009) and necessitates controlling classifiers when investigating the animacy electrophysiological effect in Mandarin Chinese.

It is important to note that the concept of lemma-level competition is based on the assumption that only the eventually selected lemma can be activated at the phonological word-form stratum. This assumption applies to Levelt’s model but not necessarily to other language production models such as interactive models (e.g., Dell, 1986, 2013) and cascaded models (e.g., Caramazza, 1997; Navarrete & Costa, 2005; Peterson & Savoy, 1998).

### Study of language production: electrophysiology

Electrophysiological evidence has long been employed by researchers to obtain time-course information during language production. Often, the N400 and P600 components are investigated.

The N400 is a negative deflection primarily centred over the centroparietal regions and observed between 250 and 500 ms after stimulus onset. It exhibits a maximum at approximately 400 ms post-stimulus onset (Kutas & Hillyard, 1980a, 1980b, 1984). It has been proposed as an indicator of semantic integration, assisting speakers in the appropriate selection of words to fit within the context. Several studies have reported larger N400 effects for naming objects under categorically unrelated against related contexts in object naming tasks (Blackford et al., 2012; Greenham et al., 2000; Wang et al., 2019). Some researchers concluded that this N400 effect was due to lemma-level competition resulting from strong conceptual-level activation (Costa et al., 2009; Wang et al., 2019).

Finally, the P600 component is a positive-going deflection centred around the centroparietal regions, having an onset around 500 ms post-stimulus onset and peaking around 600 ms post-stimulus onset (Osterhout & Holcomb, 1992). It has been proclaimed as an indicator of syntactic processing (Hagoort & Brown, 2000; Popov et al., 2020).

In summary, the N400 and P600 components have been reported as reflections for lemma-level competition in Levelt’s model. However, the N400 is semantically driven, for example, semantic categories and/or animacy, while the P600 is syntactically driven, for example, the syntactic element of classifiers (Blackford et al., 2012; Costa et al., 2009; Greenham et al., 2000; Wang et al., 2019).

### The current study

In the current study, to investigate the role of animacy in language production independent of semantic categories (Lupker, 1979; Lupker & Katz, 1981), we manipulated the congruency (congruent vs. incongruent) of animacy together with the dominant Mandarin Chinese classifier in PWI while only including inanimate–inanimate target–distractor pairs in the animacy-congruent conditions. If words sharing animacy indeed have their nodes located in closer proximity in the semantic network similar to semantic categories, as per our hypothesis, we predict the following: at the behavioural level, we predict lower naming accuracies and/or longer naming latencies under animacy-congruent conditions relative to incongruent ones at the behavioural level. Second, in accordance with the results of Wang et al. (2019), we predict a more negative amplitude between 275 and 575 ms post-stimulus onset under animacy-incongruent conditions relative to congruent conditions (i.e., an N400 effect). Regarding classifiers, we predict identical results as Wang et al. (2019) in the current study. That is, there is no behavioural dominant classifier congruency effect but a more negative amplitude in the 275–575 ms time window (i.e., an N400 effect) for classifier-incongruent against classifier-congruent conditions.

## Methods

### Participants

Thirty-three (two of which were excluded in the later analysis) native Mandarin Chinese speakers (aged 18–35) in the Netherlands gave informed consent to participate in this experiment. All participants had normal or corrected-to-normal vision, had earned (or were studying for) a university degree, and had no self-reported history of neurological/psychological impairments or language disorders. Each participant received €10 for their participation. This study was approved by the ethics committee of the Faculty of Humanities at Leiden University. The combination of number of participants and target words (i.e., number of observations per condition) was 1,302, resulting in larger statistical power than previous comparable studies (De Zubicaray et al., 2018; Gauvin et al., 2019; Wang et al., 2019).

### Materials

We selected 42 black-and-white line drawings, taken from Severens’ picture database (37 pictures) (Severens et al., 2005) or designed them ourselves (five pictures, i.e., the pictures of *doll*, *earring*, *snowman*, *dustpan*, and *plug*) that use the target nouns (disyllabic words) as their names. Every picture was displayed four times with a written distractor noun in each condition. The distractor nouns are selected on the basis of their animacy and the (dominant) Mandarin Chinese classifier congruency with the target nouns. The frequency of distractors was obtained according to the Modern Chinese Frequency Dictionary (1998) (C. R. Huang et al., 1998). The use of the (dominant) Mandarin Chinese classifiers for nouns and the number of distractor noun strokes were validated using the Xinhua dictionary (11th edition, Linguistics Institute of the Chinese Academy of Social Sciences, 2011). There was no significant difference in word frequency and visual complexity (numbers of strokes) among the four conditions; for word frequency, *F* (3, 164) = 2.1342, *p* = .10; for number of strokes, *F* (3, 164) = .5622, *p* = .64. Distractors have no orthographic or phonological relationship with the target picture names.

### Experimental design and procedure

As shown in Table 1, this experiment adopted a two-by-two full factorial within-subject within-item (where item is used to represent a target noun and hence item and subject are crossed random variables) orthogonal experimental design: animacy (A) and the dominant Mandarin Chinese classifier (C) are the two factors, while congruent (+) and incongruent (−) are the two levels. Therefore, in total, we have four conditions: A+C+, A+C−, A−C+, and A−C−. In each trial, the black-and-white picture has a distractor (from one of the four conditions) superimposed on the centre of the picture. Consequently, each participant saw the 42 images four times, resulting in 168 trials. These trials were presented pseudo-randomly.

**Table 1.** An example of a target picture presented with distractor nouns under each condition.

	Condition			
Target picture nouns	A+C+	A+C−	A−C+	A−C−
纸袋, paper bag, /zhi3dai4/ classifier, 个, /ge4/	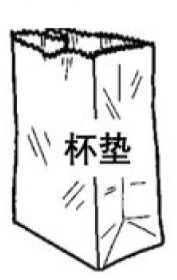	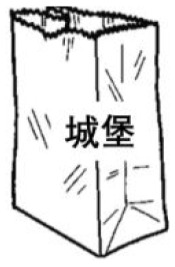	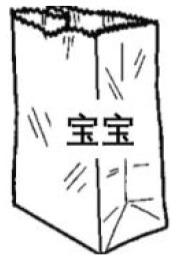	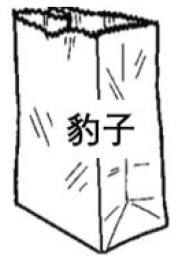
Distractor	coaster /bei1dian4/	castle /cheng2bao3/	baby /bao3bao3/	leopard /bao4zi/
Classifier of the distractor	个 /ge4/	座 /zuo4/	个 /ge4/	只 /zhi1/

The experiment comprised three successive sessions: a familiarisation session, a practice session, and an experimental session. In the familiarisation session, each picture was presented for 3 s with its target name below it. Participants were required to view the images and their corresponding target names. In the practice session, each picture was presented with the string “XXX” superimposed on the target picture. The participants were asked to produce the target name of the picture correctly and overtly while ignoring the superimposed string “XXX.” Responses that deviated from the target nouns were corrected by the experimenter after the second session had completed. In the experimental session, the 168 trials were pseudo-randomly divided into two blocks with a short break between them. The length of the break was determined by the participant. The first four trials of each block are always practice trials and not included in further analyses. In each trial, a fixation point (“+”) was presented for 300 ms, followed by a blank screen (200 ms), a picture with a distractor (3,000 ms), and finally, another blank screen (500 ms) before the subsequent trial began.

The entire task was presented by E-prime Version 2. In the experiment, participants sat in front of a computer screen in a dimly lit room and were required to name the pictures using the corresponding bare nouns as fast and accurately as possible. Vocal responses and electroencephalograms (EEG) were recorded simultaneously.

### Audio and electroencephalogram (EEG) recordings

The vocal response was recorded via E-prime using online scripts during the entire trial. EEG data were recorded via BrainVision Recorder software (Version 1.23.0001) from Brain Products GmbH. We used a 32-channel EasyCap electrode on the standard scalp sites of the comprehensive international 10/20 system. Six external electrodes were used to measure the vertical electrooculogram (VEOG, placed above and below the left eye), the horizontal electrooculogram (HEOG, placed in the external canthus of each eye), and the mastoids (placed at the mastoids). Impedance was controlled and kept below 5 kΩ, and the sampling rate was 1,024 Hz using actiCAP Control Software (Version 1.2.5.3).

### Data analysis

#### Behavioural data analysis

Trials including incorrect or incomplete vocal responses within 3,000 ms were regarded as failure to respond and excluded from further analysis. Naming latencies for correct trials were extracted from the sound recordings using Praat version 6.1.09 (Boersma, 2007) that measure the length of the time interval between target onset and voice onset (verified manually and adjusted if necessary). Trials with naming latencies larger than 3 SDs from the individual subject and item mean were excluded (1.03% of all data, 1.31% for C+A+, 1.24% for C−A+, 0.84% for C+A−, and 0.75% for C−A−). Naming accuracies and naming latencies were analysed using the *glmer()* function in the lme4 library (version 1.1-29) in *R* (version 4.1.1) with binomial and gamma (with identity link) distributions for the accuracies and latencies, respectively (Lo & Andrews, 2015). The frequency and number of strokes of distractor nouns were first mean-centred and then included as (fixed) nuisance variables to control potential confounds (Welham et al., 2014). Animacy and classifier congruency (congruent vs. incongruent) were included as sum-coded (−1 vs. 1) fixed factors. The maximal random effect structure was used as the starting model for the backward elimination strategy, where the BIC, AIC (Kuha, 2004), and/or likelihood ratio tests (Lewis et al., 2011) were employed as criteria for model selection (Bates, 2007; Bates et al., 2014). When nonconvergence or singular issues occurred, the random effect structure was simplified until the model converged (Barr et al., 2013). The model and its assumptions were checked by visualising the residuals and model predicted values.

#### EEG data analysis

##### EEG data preprocessing

We also excluded trials including incorrect or incomplete vocal responses within 3,000 ms (failed trials) in the electrophysiological data analysis. The MATLAB 2017b toolbox EEGLab 14_0_0b (Delorme & Makeig, 2004) was used for the off-line preprocessing of the EEG data, which included re-referencing, band-pass filtering, notch filtering, resampling, extracting epochs, baseline correction, bad channel interpolation, visual trial rejections, removing artefacts, and automatic trial rejection. Re-referencing was performed based on the average of both mastoid electrodes. A band-pass filter was applied from 0.1 to 30 Hz, and a notch filter was applied from 48 to 52 Hz to decrease power line noise interference (Ahmad et al., 2012). Resampling was performed from 1,024 to 256 Hz to compare with previous studies (i.e., Wang et al., 2019). Epochs from −200 to 700 ms were computed with −200 to 0 ms prestimulus intervals as baseline correction. Interpolation was carried out on noisy channels. Visual trial rejection was used to remove trials with noisy or large fluctuations in amplitude. Removing artefacts was performed via ICA to remove possible noise sources, including cardiac signals, muscle contraction, and eyeball movement. ADJUST v 1.1.1 was used to recognise these types of noise (Mognon et al., 2011). Finally, automatic trial rejection was performed on trials with an amplitude of more than ±100μV. Participants with more than 2/3 rejected trials were not included for further analysis. As a result, we had 31 participants for further analysis.

##### A priori amplitude analyses

We conducted statistical modelling on a priori selected time windows and electrodes based on previous literature (i.e., Wang et al., 2019). They are electrodes F3, FC1, FC5, C3, CP1, CP5, P3, PO3, F4, FC2, FC6, C4, CP2, CP6, P4, and PO4 in the 275–575 ms time window. In greater detail, we grouped the electrodes according to their location, that is, left parietal central, left frontal central, right parietal central, and right frontal central. The *lmer()* function in the lme4 1.1-29 library in R version 4.1.1 was used for statistical modelling, and that of the lmerTest version 3.1-3 library was used to obtain the p-values. The amplitudes at 275–575 ms on F3, FC1, FC5, C3, CP1, CP5, P3, PO3, F4, FC2, FC6, C4, CP2, CP6, P4, and PO4 were included as response variables. The location of electrodes, that is, left parietal central, left frontal central, right parietal central, and right frontal central, was included in the fixed variables. Otherwise, the statistical modelling is the same as that previously described in the “Behavioral data analysis” section.

##### Exploratory permutation-based cluster mass analyses (−200 to 700 ms)

In addition, a mass univariate cluster permutation test was performed to explore the full temporo-spatial extent of animacy effects. First, a permutation test for a linear mixed model with threshold-free cluster enchantment (TFCE) as type I error correction was conducted (E = 0.66, *H* = 2, see Smith & Nichols, 2009) to identify time windows and channels that detect an effect across the combined four levels of the two main effects (Visalli et al., 2024). The formula of the linear mixed model in the permutation test is Amplitude ~ Number of Stroke + Frequency of distractor + Condition (the combined four levels of the two main effects) + (1 | participant) + (1 | item). The family-wise error for the cluster permutation test was set at 5%. It is important to note that, in this study, the final few milliseconds pose confounding issues because of the overlap between the articulation and manipulated conditions. Therefore, empirical distribution of the TFCE values was obtained with the peaked values from before 500 ms post stimulus onset (Smith & Nichols, 2009), which was the lower bound of naming latencies, to avoid motor artefacts from articulation. With the time window and electrodes obtained from the mass univariate cluster permutation, a linear mixed model will be performed for follow-up analysis, as explained in the “A priori amplitude analyses” section.

## Results

### Behavioural data analysis results

The error rates for each condition are as follows: 4.08% (C−A−), 3.71% (C−A+), 3.14% (C+A−), and 3.68% (C+A+). The average error rate across the four conditions is 3.65%. The descriptive results are shown in the online Supplemental Material.

Regarding naming accuracies, as presented in Table 2, a generalised (binomial) mixed-effects model showed neither an effect of animacy (*β* = 0.014, 95% CI = [−0.138, 0.166], *SE* = 0.077, *z* = 0.182, *p* = .855) nor of classifier (*β* = −0.080, 95% CI = [−0.233, 0.072], *SE* = 0.077, *z* = −1.034, *p* = .301) nor an interaction effect (*β* = −0.072, 95% CI = [−0.220, 0.077], *SE* = 0.076, *z* = −0.947, *p* = .343).

**Table 2.** Detailed information on the best fitting model for naming accuracies.

Formula: Naming accuracies ~ Number of strokes + Frequency of the distractor + Animacy congruency (congruent vs. incongruent) × Classifier congruency (congruent vs. incongruent) + (1 | subject) + (1 | item)				
Fixed effects	Estimate	95% CI [low, high]	z-value	Pr (>|*z*|)
(Intercept)	4.213	[3.047, 5.057]	8.859	<0.001
Number of strokes	0.010	[−0.176, 0.196]	−0.106	0.916
Frequency of the distractor	−0.030	[−0.172, −0.232]	−0.294	0.769
Animacy incongruent	0.014	[−0.138, 0.166]	0.182	0.014
Classifier incongruent	−0.08045	[−0.234, 0.072]	−1.034	0.301
Animacy incongruent: Classifier incongruent	−0.072	[−0.077, 0.0220]	−0.947	0.343
Random effects				
$\sigma^{2}$	1.000			
$\tau_{item}$	1.389			
$\tau_{participant}$	0.874			
$N_{item}$	42			
$N_{subject}$	31			
ICC	1.000			
Observations	4,653			
Marginal $R^{2}$ /Conditional $R^{2}$	0.020/1.000			

**Figure 1.**
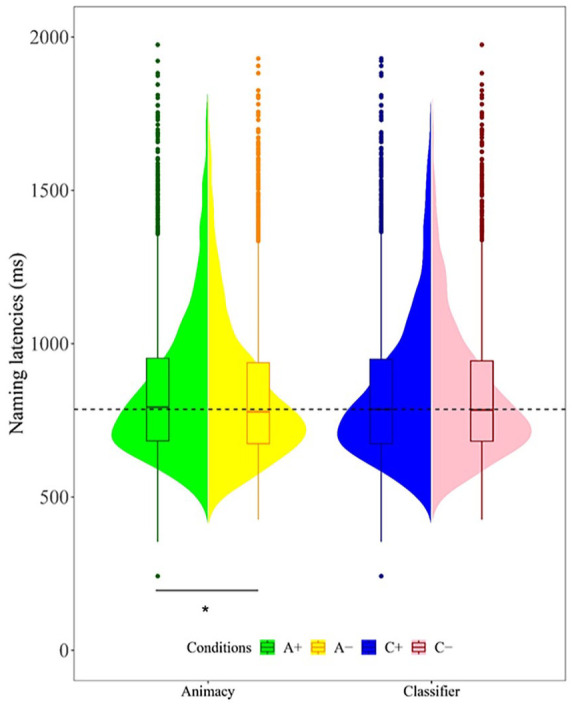
Naming latencies across the conditions of animacy and classifier congruency.

As for the naming latencies for correct responses, as shown in Figure 1 and Table 3, a generalised linear mixed-effects model with the gamma distribution and identity link showed that animacy-incongruent conditions exhibit statistically shorter naming latencies than congruent conditions (*β* = −5.641, 95% CI = [1.712, 21.015], *SE* = 2.555, *z* = −2.208, *p* = .0273), but neither the classifier (*β* = −0.681, 95% CI = [−6.661, 9.548], *SE* = 4.135, *z* = −0.266, *p* = .7901) nor the interaction (*β* = −0.041, 95% CI = [−12.418, 12.091], *SE* = 2.47091, *z* = −0.017, *p* = .980) effect are significant.

**Table 3.** Detailed information on the best fitting model for naming latencies.

Formula: Naming latencies ~ Number of strokes + Frequency of distractor + Animacy congruency (congruent vs. incongruent) × Classifier congruency (congruent vs. incongruent) + (1 − subject) + (1 − item)				
Fixed effects	Estimate	95% CI [low, high]	*t*-value	Pr(>|*z*|)
(Intercept)	904.275	[887.335, 908.492]	104.471	<0.001
Number of strokes	−1.397	[−7.027, 4.233]	−0.486	0.627
Frequency of the distractor	−6.765	[−12.031, −1.499]	−2.518	0.012 *
Animacy incongruent	−5.6418	[−10.649, −0.632]	−2.208	0.028 *
Classifier incongruent	−0.6814	[−5.692, 4.331]	−0.266	0.7901
Animacy incongruent: Classifier incongruent	−0.041	[−4.884, 4.801]	−0.017	0.9867
Random effects				
$\sigma^{2}$	0.057			
$\tau_{item}$	43.617			
$\tau_{subject}$	48.643			
$N_{item}$	42			
$N_{subject}$	31			
ICC	1.000			
Observations	4,653			
Marginal $R^{2}$ /Conditional $R^{2}$	0.020/1.000			

### EEG data analysis results

#### Planned analyses

The amplitude for a priori selected channels in the 275–575 ms time window shows that (a) animacy-incongruent conditions have a significantly more negative amplitude compared with congruent conditions (*β* = −0.047, 95% CI = [−0.053, −0.041], *SE* = 0.003, *t* = −14.292, df = 5,359,000, *p* < .001); (b) classifier-incongruent conditions have a significantly more positive amplitude relative to the congruent conditions (*β* = 0.034, 95% CI = [0.028, 0.041], *SE* = 0.003, *t* = 10.588, df = 5,535,000, *p* < .001); and (c) a significant interaction effect between animacy and classifier (*β* = −0.007, 95% CI = [−0.013, −0.001], *SE* = 0.003, *t* = −2.217, df = 5,551,000, *p* = .027).

**Figure 2.**
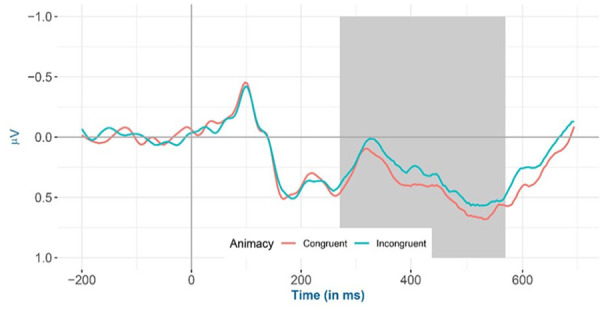
Mean amplitude of all selected electrodes (F3, FC1, FC5, C3, CP1, CP5, P3, PO3, F4, FC2, FC6, C4, CP2, CP6, P4, and PO4) for animacy-congruent versus animacy-incongruent conditions from −200 to 700 ms after stimulus onset, shadowed in 275–575 ms.

**Figure 3.**
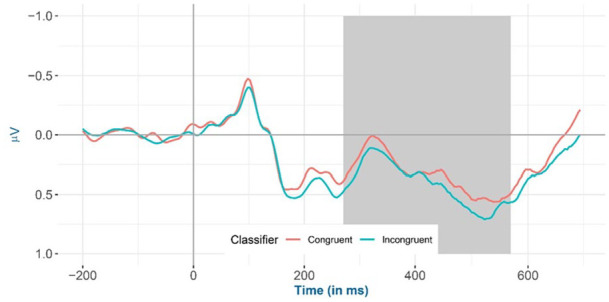
Mean amplitude of all selected electrodes (F3, FC1, FC5, C3, CP1, CP5, P3, PO3, F4, FC2, FC6, C4, CP2, CP6, P4, and PO4) for classifier congruent versus incongruent conditions from −200 to 700 ms after stimulus onset, shadowed in 275–575 ms.

**Figure 4.**
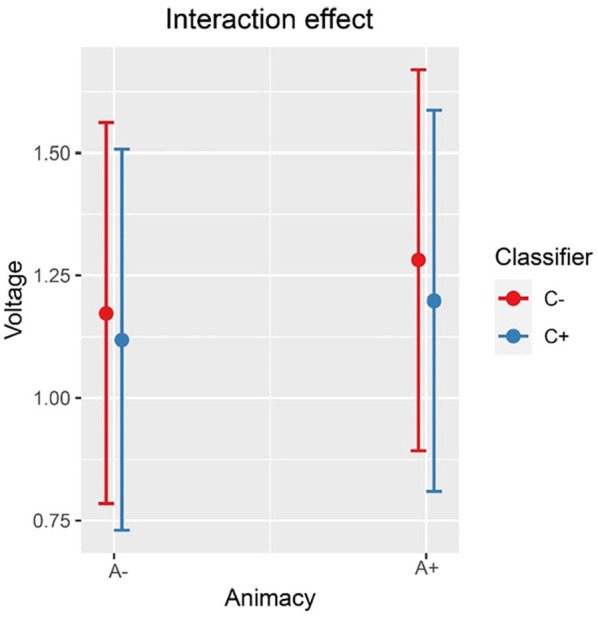
Interaction effect between animacy and classifiers on the amplitude of all selected electrodes (F3, FC1, FC5, C3, CP1, CP5, P3, PO3, F4, FC2, FC6, C4, CP2, CP6, P4, and PO4) at 275–575 ms.

Inspection of the means predicted by the model revealed that (see Figure 2 and Table 4) the mean amplitude for the animacy-incongruent conditions (estimated marginal *M*: 1.265 μV) is more negative than that for the animacy-congruent conditions (estimated marginal *M*: 1.359 μV). Regarding the classifier-congruency effect (see Figure 3 and Table 4), the estimated marginal mean amplitude for the incongruent conditions (1.346 μV) is more positive than that for the classifier-congruent conditions (1.278 μV). Regarding the interaction, inspection of the estimated cell means (Figure 4 and Table 4) showed that the classifier-congruency effect was larger when animacy was congruent compared with when animacy was incongruent.

**Table 4.** Result for the time window 275–575 ms for electrodes F3, FC1, FC5, C3, CP1, CP5, P3, PO3, F4, FC2, FC6, C4, CP2, CP6, P4, and PO4.

Formula: Amplitude ~ Number of strokes + Frequency of distractor + Location (left parietal central vs. left frontal central vs. right parietal central vs. right frontal central) + Animacy congruency (congruent vs. incongruent) × Classifier congruency (congruent vs. incongruent) + (1 − participant) + (1 − item)				
Fixed effects	Estimate	95% CI [low, high]	t-value	Pr(>|*t*|)
(Intercept)	1.312	[0.911, 1.713]	6.606	< 0.001
Number of strokes	−0.052	[−0.059, −0.045]	−13.761	< 0.001 ***
Frequency of the distractor	−0.029	[−0.037, −0.021]	−7.395	< 0.001 ***
left frontal central	−1.707	[−1.725, −1.689]	−188.952	< 0.001 ***
right parietal central	0.005	[−0.012, 0.023]	0.606	0.5448
right frontal central	−1.7	[−1.718, −1.682]	−188.159	< 0.001 ***
Animacy incongruent	−0.047	[−0.053, −0.041]	−14.292	< 0.001***
Classifier incongruent	0.034	[0.028, 0.041]	10.588	< 0.001***
Animacy incongruent: Classifier incongruent	−0.007	[−0.013, −0.001]	−2.217	0.0266 *
Random effects				
$\sigma^{2}$	56.695			
$\tau_{item}$	0.192			
$\tau_{subject}$	1.090			
$N_{item}$	41			
$N_{subject}$	31			
ICC	0.021			
Observations	5,558,336			
Marginal $R^{2}$ /Conditional $R^{2}$	0.013/0.033			

#### Results of exploratory permutation-based TFCE analyses

**Figure 5.**
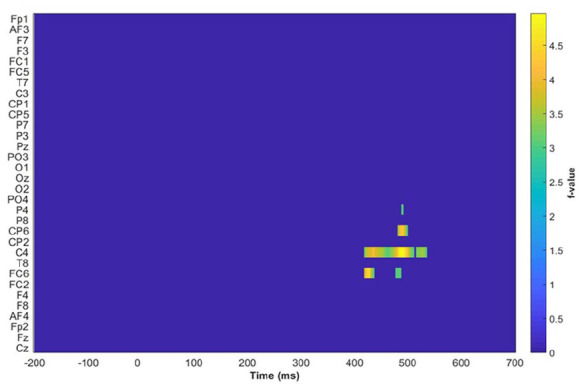
Results of the permutation test across all electrodes from −200 to 700 ms after stimulus onset. The highlighted regions are CP6, C4, and FC6 in the time window of 400–500 ms.

A mass univariate cluster permutation test was performed using a linear mixed model (Amplitude ~ Number of Stroke + Frequency of distractor + Conditions) (the combined four levels of the two main effect) + (1 − participant) + (1 − item) and TFCE to control the family-wise error at 5% (shown in Figure 5). The significant cluster reported in the mass univariate cluster permutation test occurs in the centroparietal area and spans 400–500 ms after stimulus onset. On this basis, CP6, C4, and FC6 were selected for further analysis.

For the cluster highlighted in Figure 5, the mixed model yielded the same result in the planned analysis. That is, (a) animacy-incongruent conditions have a significantly more negative effect compared with animacy-congruent conditions (*β* = −0.197, 95% CI = [−0.223, −0.172], *SE* = 0.013, *t* = −15.158, df = 325,500, *p* < .001); (b) classifier-incongruent conditions have a significantly more positive effect relative to the congruent conditions (*β* = 0.162, 95% CI = [0.136, 0.187], *SE* = 0.013, *t* = 12.515, df = 328,800, *p* < .001); and (c) a significant interaction effect between animacy and classifier (*β* = −0.053, 95% CI = [−0.078, −0.028], *SE* = 0.013, *t* = −4.137, df = 329,000, *p* < .001).

**Figure 6.**
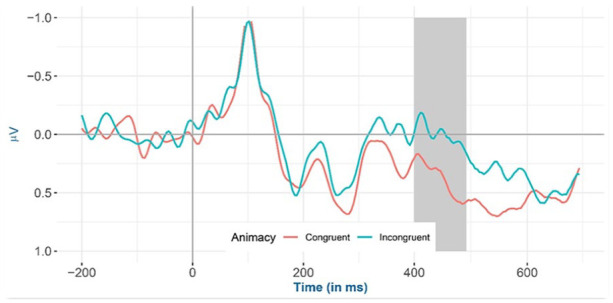
Mean amplitude for all selected channels (CP6, C4, and FC6) for animacy-congruent versus animacy-incongruent conditions from −200 to 700 ms after stimulus onset (the highlighted time window is between 400 and 500 ms) in the exploratory analysis.

**Figure 7.**
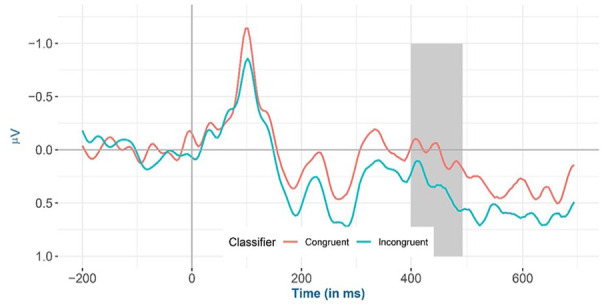
Mean amplitude for all selected channels (CP6, C4, and FC6) for classifier congruent versus incongruent conditions from −200 to 700 ms after stimulus onset (the highlighted time window is between 400 and 500 ms) in the exploratory analysis.

**Figure 8.**
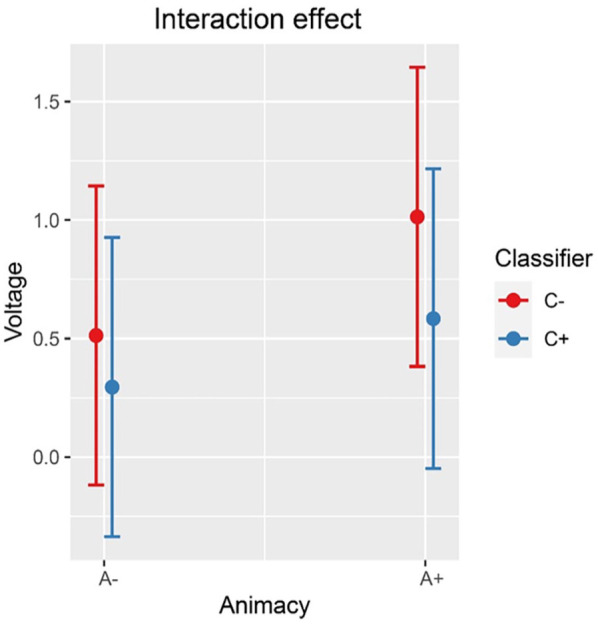
Interaction effect between animacy and classifier congruency on the amplitude of all selected channels (CP6, C4, and FC6) at 400–500 ms after stimulus onset in the exploratory analysis.

Inspection of the means predicted by the model revealed that (see Figure 6 and Table 5), the mean amplitude for animacy-incongruent conditions is (estimated marginal *M*: 0.574 μV) more negative than that for animacy-congruent conditions (estimated marginal *M*: 0.964 μV). Regarding the classifier congruency effect (see Figure 7 and Table 5), the estimated marginal mean amplitude for incongruent conditions (0.929 μV) is more positive than that for classifier-congruent conditions (0.605 μV). Regarding the interaction, inspection of the estimated cell means (see Figure 8 and Table 5) showed that the classifier incongruency effect was larger when animacy was congruent than when it was incongruent.

**Table 5.** Mixed model result in the time window between 400 and 500 ms for channels CP6, C4, and FC6 in the exploratory analysis.

Formula: Amplitude ~ Frequency of distractor + Location (left frontal central vs. right parietal central) + Animacy congruency (congruent vs. incongruent) × Classifier congruency (congruent vs. incongruent) + (1 − participant) + (1 − item)				
Fixed effects	Estimate	95% CI [low, high]	t-value	Pr(>|*t*|)
(Intercept)	0.767	[0.110, 1.425]	2.351	0.024 *
Frequency of the distractor	−0.041	[−0.071, −0.010]	−2.606	0.009 **
Location right parietal central	−1.319	[−1.372, −1.266]	−48.813	< 0.001 ***
Animacy incongruent	−0.197	[−0.223, −0.172]	−15.158	< 0.001 ***
Classifier incongruent	0.162	[0.136, 0.187]	12.515	< 0.001***
Animacy incongruent: Classifier incongruent	−0.053	[−0.078, −0.028]	−4.137	< 0.001 ***
Random effects				
$\sigma^{2}$	53.376			
$\tau_{item}$	0.414			
$\tau_{subject}$	1.753			
$N_{item}$	41			
$N_{subject}$	31			
ICC	0.057			
Observations	329,112			
Marginal $R^{2}$ /Conditional $R^{2}$	0.008/0.065			

## Discussion

To summarise, we observed a small animacy congruency effect but no classifier congruency effect at the behavioural level. Furthermore, at the electrophysiological level, a larger negativity was observed for animacy-incongruent conditions relative to congruent conditions. In contrast, for classifiers, a positivity was found for incongruent conditions compared with congruent conditions. An electrophysiological interaction effect between animacy and the dominant Mandarin Chinese classifier was also observed, that is, the classifier incongruency effect was larger (more positive) when animacy was congruent rather than incongruent.

### Animacy interference effect

Before discussing the implications of our findings for Levelt’s model of speech production, it is worth reiterating that previous research on animacy interference from the perspective of semantic networks in word production is largely non-existent to the best of our knowledge. The closest existing literature resembling animacy interference includes studies regarding a different semantic feature, namely shape. In those studies (De Zubicaray et al., 2018; Gauvin et al., 2019), the authors reported shape interference effects associated with reduced activity in the left posterior middle temporal gyrus (pMTG) for shape-congruent conditions relative to incongruent conditions. As the pMTG area has reliably been observed to be activated during lexical level processing in language production (Binder et al., 2009; Cabeza & Nyberg, 2000; Indefrey, 2011; Indefrey & Levelt, 2004; Vigneau et al., 2006), the authors concluded that shape plays a similar role as semantic categories in word production, that is, being processed at the conceptual level and activating lexical entries at the lemma level in Levelt’s model.

In the current study, the observed animacy interference effect was accompanied by a more negative amplitude under animacy-incongruent conditions relative to congruent conditions. The negative amplitude was maximal at the parietal and central electrodes with a peak around 400 ms after stimulus onset, resembles a classical N400 effect (Kutas & Hillyard, 1980a, 1980b, 1984). As discussed in the introduction, the N400 effect is reliably observed to be more negative for incongruent against congruent conditions in electrophysiological studies on language production (Blackford et al., 2012; Costa et al., 2009; Debruille et al., 2008; Greenham et al., 2000; Kutas & Federmeier, 2011; Wang et al., 2019) and has been attributed to lemma-level competition originating at the conceptual level (S. Huang & Schiller, 2021; Wang et al., 2019; but see Blackford et al., 2012). To reiterate, lemma-level competition occurs due to multiple concepts being activated, and activation passed down to the lemma level, resulting in multiple lemmas becoming activated and subsequently competing for selection. Thus, we assume here that the animacy congruency effect that we observed results from lemma-level competition in Levelt’s model. Consequently, we conclude that the semantic interference effects previously reported for semantic category congruency (Bürki et al., 2020; S. Huang & Schiller, 2021; Wang et al., 2019) are at least partially caused by more elementary semantic features such as animacy.

Various models have been proposed to explain category-specific effects in naming tasks. On the one hand, concepts congruent in animacy could be represented by nodes in a semantic network and have their nodes in closer proximity than shape-incongruent words in the semantic network as compared with concepts incongruent in animacy (Collins & Loftus, 1975; Roelofs, 1992, 1993, 1996). On the other hand, it has been argued that such effects could be explained by overlap in elementary features among living and non-living entities (Humphreys & Forde, 2001; see also McRae et al., 2005). Finally, the role of semantic features is not mutually exclusive with the role of categories (Levelt, 1999; Levelt et al., 1999; Roelofs, 1996). As, in the present study, the animacy-congruent and incongruent conditions were confounded with features (e.g., see McRae et al., 2005); both types of models can account for the effect of animacy congruency.

### Dominant classifier effect and its interaction with animacy

Because the animacy of a given noun determines the choice of its classifiers (11th edition, Linguistics Institute of the Chinese Academy of Social Sciences, 2011), and Mandarin Chinese classifiers have been reported to affect electrophysiological responses in naming studies (Wang et al., 2019), we were required to control for this variable. However, because research on the role of classifier congruency in language production is also still scarce, our results provide interesting insights into classifier effects. We found (a) a more *positive* amplitude for classifier-incongruent against classifier-congruent conditions and (b) the classifier incongruency effect became larger (more positive) when animacy was congruent than when it was incongruent. This result contradicts the results reported by Wang et al., that is, a more *negative* (purportedly N400) effect for the classifier-incongruent condition (Wang et al., 2019). Importantly, Wang et al. conducted a permutation test over the full temporo-spatial extent of the EEG signal and did not observe any P600 effects.

To address this discrepancy, it is important to look more closely at the N400 and P600 components. As discussed in the introduction, in language production, the N400 effect is semantically driven (Ganushchak et al., 2011; Krott et al., 2019; Wang et al., 2019), whereas the P600 effect is presumably syntactically driven (Hagoort & Brown, 2000; Popov et al., 2020). Given that classifiers have lexico-syntactic properties, the more positive amplitude observed in the current study could reflect a P600 effect. Therefore, we posit that the more negative N400 effect of classifier congruency observed by Wang et al. might be semantically driven because animacy was covarying with the classifier congruency effect and not adjusted for in their study, in contrast to the present study. We further hypothesise that the more positive P600 effect observed in the current study is instead syntactically driven. This P600 effect could be observed in the present study because the classifier congruency effect occurred under conditions controlled for animacy congruency and thus was presumably more representative of its lexico-syntactic properties.

Closer inspection of the differences in material between the present study and Wang et al. (2019) supports this assumption. The ratio of animacy-congruent/incongruent (i.e., animacy overlap) target–distractor pairs under classifier congruent conditions was 83.33% in Wang et al. (2019) versus 50% in the current study. For classifier-incongruent conditions, the ratio of animacy-congruent/incongruent target–distractor pairs was 86.67% in Wang et al. (2019) vs. 50% in the current study. Hence, the classifier congruency effect would be biased towards the conditions where animacy is congruent, resulting in an N400 effect and masking the syntactically driven P600 effect in Wang et al. (2019). Therefore, a greater overlap in semantic features between target and distractor words might result in concurrent semantic processing of classifiers (Rose et al., 2019; Vieth et al., 2014, but see Mahon et al., 2007), resulting in an N400. In the current study, classifier congruency was less confounded with semantic feature overlap than in Wang et al. (2019), reducing concurrent semantic processing and unmasking the syntactically driven processing of classifiers.

In other words, given the interaction effect between animacy and classifier congruency in ERP amplitudes and the negative main animacy effect being larger than the positive main classifier electrophysiological effect in the current study, we argue that the syntactically driven P600 effect of classifiers in the Wang et al. study might have been masked by the semantically driven N400 effect of animacy. However, the exact manner in which classifiers and semantic features interact should be investigated in future studies.

## Conclusion

In conclusion, the current study underscores the role of animacy in word production. Concepts that differ in animacy have been shown to differ in overlap of semantic features (McRae et al., 2005). Thus, in terms of Levelt’s model of speech production (Levelt, 1999), the effects of animacy in PWI tasks could be explained by animacy-congruent words being activated more strongly at the conceptual level and subsequently at the lemma level, where animacy-congruent lexical candidates compete for selection.

Furthermore, when the animacy effect is controlled for in investigating the Mandarin Chinese classifier congruency effect, a positivity rather than negativity was observed, suggesting that, that is, classifiers are primarily processed syntactically at the lemma level. As semantic features are not possible to be fully controlled when investigating the classifier congruency effect experimentally, observation data with proper statistical methods (e.g., causal inference method) could be used to further investigate the nature of the classifier congruency effect.

## Supplemental Material

sj-docx-1-qjp-10.1177_17470218241281868 – Supplemental material for The role of animacy in language production: evidence from bare noun naming Supplemental material, sj-docx-1-qjp-10.1177_17470218241281868 for The role of animacy in language production: evidence from bare noun naming by Yufang Wang, Jurriaan Witteman and Niels O Schiller in Quarterly Journal of Experimental Psychology
