# Supplementary material for: The role of animacy in language production: evidence from bare noun naming
Source: Q J Exp Psychol (Hove). 2024 Sep 28;78(7):1461–73. doi: 10.1177/17470218241281868 (PMC12181646; doi:10.1177/17470218241281868)
Supplement: sj-docx-1-qjp-10.1177_17470218241281868 – Supplemental material for The role of animacy in language production: evidence from bare noun naming [file sj-docx-1-qjp-10.1177_17470218241281868.docx]

Supplementary Material for:

**The role of animacy in language production: evidence from bare noun naming**

Yufang Wang ^a, b^, Jurriaan Witteman ^a, b^, & Niels O. Schiller ^a, b, c^

^a^ Leiden University Center for Linguistics, Leiden University, The Netherlands,

^b^ Leiden Institute for Brain and Cognition, Leiden University, The Netherlands

^c^ Department of Linguistics and Translation, City University of Hong Kong, Hong Kong SAR

**Supplementary Material A. Descriptive results for naming accuracies and naming latencies in the four conditions**

| Condition | Naming accuracies (%) | | Naming latencies (ms) | |
| --- | --- | --- | --- | --- |
|  | Mean | SD | Mean | SD |
| C-A- | 96 | 4.076 | 837 | 235.318 |
| C-A+ | 96 | 2.840 | 848 | 235.440 |
| C+A- | 97 | 2.989 | 842 | 248.386 |
| C+A+ | 96 | 3.853 | 855 | 248.057 |

**Supplementary Material B. Stimuli used in the experiment**

| Target noun | Dominant classifier | Distractor type | | | |
| --- | --- | --- | --- | --- | --- |
|  |  | Dominant classifier congruent | | Dominant classifier incongruent | |
|  |  | Animacy congruent | Animacy incongruent | Animacy congruent | Animacy incongruent |
| 钱包  qian2bao1  wallet | 个  ge4 | 本子  ben3zi  notebook | 水手  shui3shou3  sailor | 彩虹  cai3hong2  rainbow | 蝴蝶  hu2die2  butterfly |
| 拖鞋  tuo1xie2  slipper | 只  zhi1 | 踏板  ta4ban3  pedal | 乌龟  wu1gui1  turtle | 大衣  da4yi1  overcoat | 娘子  niang2zi3  wife |
| 皮球  pi2qiu2  ball | 个  ge4 | 桃子  tao2zi  peach | 士兵  shi4bing1  soldier | 图书  tu2shu1  books | 公鸡  gong1ji1  rooster |
| 包裹  bao1guo3  package | 个  ge4 | 脑袋  nao3dai  head | 新娘  xin1niang2  bride | 单车  dan1che1  bicycle | 来客  lai2ke4  visitor |
| 水桶  shui3tong3  bucket | 只  zhi1 | 风筝  feng1zheng1  kite | 蚯蚓  qiu1yin3  earthworm | 云朵  yun2duo4  clouds | 女工  nv3gong1  female worker |
| 柜子  gui4zi  cabinet | 只  zhi1 | 蛋黄  dan4huang2  yolk | 小鸟  xiao3niao3  little bird | 书桌  shu1zhuo1  desk | 姐姐  jie3jie3  sister |
| 杯子  bei1zi  cup | 只  zhi1 | 柑橘  gan1ju2  tangerine | 天鹅  tian2e2  swan | 牙刷  ya2shua1  toothbrush | 难民  nan4ming2  refugee |
| 太阳  tai4yang2  sun | 个  ge4 | 背包  bei2bao1  backpack | 小孩  xiao3hai2  child | 内衣  nei4yi1  underwear | 斑鸠  ban1jiu1  turtledove |
| 信封  xin4feng1  envelope | 个  ge4 | 篮子  lan2zi  basket | 厨师  chu2shi1  cook | 凤梨  feng4li2  pineapple | 老鹰  lao3ying1  eagle |
| 毛巾  mao2jin1  towel | 条  tiao2 | 裙子  qun2zi  skirt | 鲨鱼  sha1yu2  shark | 火箭  huo3jian4  rocket | 理事  li3shi4  director |
| 马桶  ma3tong3  toilet | 只  zhi1 | 布袋  bu4dai4  cloth bag | 雄蝶  xiong2die2  male butterfly | 磁带  ci2dai4  tape | 表哥  biao3ge1  cousin |
| 手套  shou3tao4  gloves | 只  zhi1 | 球拍  qiu4pai1  racket | 兔子  tu2zi2  rabbit | 雨伞  yu3san3  umbrella | 猎人  lie2ren4  hunter |
| 笼子  long2zi  cage | 个  ge4 | 瞳孔  tong2kong3  pupil | 保姆  bao3mu3  nanny | 轿车  jiao4che1  car | 瓢虫  piao2chong2  ladybug |
| 灯泡  deng1pao2  light bulb | 只  zhi1 | 牛角  niu2jiao3  horns | 燕子  yan4zi  swallow | 桌子  zhuo1zi  table | 律师  lv4shi1  lawyer |
| 花瓶  hua1ping2  vase | 个  ge4 | 书包  shu1bao1  bag | 保镖  bao3biao1  bodyguard | 门票  men2piao4  tickets | 情侣  qing2lv3  lovers |
| 哨子  shao4zi  whistle | 只  zhi1 | 鼻子  bi2zi  nose | 蜈蚣  wu2gong1  centipede | 面包  mian4bao1  bread | 校友  xiao2you3  alumni |
| 贝壳  bei4ke2  shell | 只  zhi1 | 筷子  kuai4zi  chopstick | 蜘蛛  zhi1zhu1  spider | 新月  xin1yue4  new moon | 骆驼  luo4tuo2  camel |
| 领带  ling3dai4  Chopsticks | 条  tiao2 | 长裤  chang2ku4  pants | 好汉  hao3han4  brave man | 监狱  jian1yu4  jail | 司机  si1ji1  driver |
| 桃子  tao2zi4  peach | 只  zhi1 | 火把  huo3ba3  torch | 蜥蜴  xi1yi4  lizard | 钢琴  gang1qin2  piano | 女王  nv3wang3  queen |
| 耳环  er3huan2  earring | 只  zhi1 | 鼻孔  bi2kong3  nostril | 蜜蜂  mi4feng1  bee | 步枪  bu4qiang1  rifle | 牧师  mu4shi1  priest |
| 袋子  dai4zi  bag | 个  ge4 | 杯垫  dai4zi  coaster | 宝宝  bao3bao3  baby | 城堡  cheng2bao3  castle | 豹子  bao4zi  leopard |
| 鞋子  xie2zi4  shoe | 只  zhi1 | 臂膀  bi4bang3  arm | 猫咪  mao1mi1  cat | 钻石  zuan4shi2  diamond | 警察  jing3cha2  policeman |
| 链子  lian4zi  chain | 条  tiao2 | 绳子  sheng3zi  rope | 毒蛇  du2she2  viper | 白云  bai4yun2  white cloud | 海豹  hai3bao4  seal |
| 气球  qi4qiu2  balloon | 只  zhi1 | 喇叭  la3ba2  horn | 鸵鸟  tuo2niao3  ostrich | 宝剑  bao3jian4  sword | 男人  nan2ren2  man |
| 雪人  xue3ren2  snowman | 个  ge4 | 瓶子  ping2zi4  bottle | 绑匪  bang3fei3  kidnapper | 蒲葵  pu2kui2  palmetto | 斑马  ban1ma3  zebra |
| 徽章  hui1zhang1  badge | 只  zhi1 | 手表  shou3biao2  watch | 袋鼠  dai4shu3  kangaroo | 铜像  tong2xiang4  bronze statue | 奴隶  nu4li4  slave |
| 爪子  zhua3zi  paw | 只  zhi1 | 镯子  zhuo2zi  bracelet | 蚊子  wen2zi  mosquito | 轮椅  lun2yi3  wheelchair | 编剧  bian1ju4  screenwriter |
| 簸箕  bo4ji2  dustpan | 个  ge4 | 礼盒  li3he2  gift box | 企鹅  qi2e2  penguin | 礼服  li3fu2  dress | 女孩  nv3hai2  girl |
| 马路  ma3lu2  road | 条  tiao2 | 围巾  wei2jin1  scarf | 鲤鱼  li3yu2  carp | 吉他  ji2ta1  guitar | 歹徒  dai3tu2  gangster |
| 轮子  lun2zi4  wheel | 只  zhi1 | 本子  ben3zi4  notebook | 老虎  lao3hu3  tiger | 灯塔  deng1ta3  lighthouse | 明星  ming2xing1  star |
| 奖杯  jiang3bei1  trophy | 只  zhi1 | 辫子  bian4zi2  braid | 蚂蚁  ma3yi3  ant | 吊桥  diao4qiao2  suspension bridge | 法官  fa3guan1  judge |
| 盘子  pan2zi4  plate | 个  ge4 | 豆荚  dou4jia2  pod | 海盗  hai3dao4  pirate | 毛衣  mao2yi1  sweater | 绵羊  mian2yang2  sheep |
| 篮子  lan2zi  basket | 只  zhi1 | 瞳孔  tong2kong3  pupil | 犀牛  xi1niu2  rhino | 坦克  tan3ke2  tank | 疯子  feng1zi  lunatic |
| 内裤  nei4ku4  underpants | 条  tiao2 | 河流  he2liu2  river | 虫子  chong2zi  insect | 爆竹  bao4zhu2  firecracker | 骑士  qi2shi4  knight |
| 玩偶  wan2ou3  doll | 个  ge4 | 泥巴  ni2ba1  mud | 胎儿  tai1er2  fetus | 口红  kou3hong2  lipstick | 猴子  hou2zi  monkey |
| 短裙  duan3qun2  skirt | 条  tiao2 | 板凳  ban3deng4  bench | 鲸鱼  jing1yu2  whale | 棒球  bang4qiu2  baseball | 海豚  hai4tun2  dolphin |
| 插头  cha2tou2  plug-in | 个  ge4 | 汉堡  han4bao3  hamburger | 天才  tian1cai2  genius | 花瓣  hua1ban4  petal | 螳螂  tang2lang2  praying mantis |
| 耙子  pa2zi  rake | 个  ge4 | 饺子  jiao3zi  dumpling | 逃兵  tao2bing1  deserter | 花灯  hua1deng1  lantern | 堂妹  tang2mei4  cousin |
| 勺子  shao2zi4  spoon | 只  zhi1 | 酒杯  jiu3bei1  wine glass | 海鸥  hai3ou1  seagull | 花轿  hua1jiao4  sedan chair | 乞丐  qi3gai4  beggar |
| 小号  xiao3hao4  trumpet | 只  zhi1 | 葫芦  hu2lu  gourd | 海龟  hai3gui1  sea turtle | 滑板  hua2ban3  skate board | 太后  tai4hou4  queen |
| 轮胎  lun2tai2  tire | 条  tiao2 | 毯子  tan3zi  blanket | 狐狸  hu2li2  fox | 化石  hua4shi2  fossil | 海狮  hai3shi1  sea lion |
| 项链  xiang4lian4  necklace | 条  tiao2 | 血管  xie3guan3  vessel | 金鱼  jin1yu2  goldfish | 画笔  hua4bi3  brush | 蟋蟀  xi1shuai4  cricket |
